# EXPRESSION OF CONCERN: Long Non‐coding RNA Urothelial Cancer‐associated 1 Promotes Bladder Cancer Cell Migration and Invasion by Way of the hsa‐miR‐145–ZEB1/2–FSCN1 Pathway

**DOI:** 10.1111/cas.70241

**Published:** 2025-10-29

## Abstract

**EXPRESSION OF CONCERN**: XueM. , PangH. , LiX. , LiH. , PanJ. and ChenW. , “Long Non‐coding RNA Urothelial Cancer‐associated 1 Promotes Bladder Cancer Cell Migration and Invasion by Way of the hsa‐miR‐145–ZEB1/2–FSCN1 Pathway,” Cancer Science 107, no. 1 (2016): 18–27, 10.1111/cas.12844.26544536 PMC4724815

This Expression of Concern is for the above article, published online on 06 November 2015 in Wiley Online Library (wileyonlinelibrary.com), and has been issued by agreement between the journal Editor‐in‐Chief, Masanori Hatakeyama; the Japanese Cancer Association; and John Wiley & Sons Australia Ltd. The Expression of Concern has been agreed upon due to several instances of image duplication identified between Figures 3b, 3f and 3 g of this article and images published in another paper by a different group of authors. These duplicated images were used to represent different scientific contexts. Although this article was published earlier, the authors did not respond to requests for comments or original data, therefore the authenticity of the images could not be verified. Furthermore, concerns were raised regarding Figures 2c, 2e, 4f and 5 g, where the western blot backgrounds appear unusually clean, deviating from typical experimental results. Due to the nature of these concerns and the absence of author response, the journal has decided to issue an Expression of Concern to inform and alert readers.

